# MitoTEMPOL Inhibits ROS-Induced Retinal Vascularization Pattern by Modulating Autophagy and Apoptosis in Rat-Injected Streptozotocin Model

**DOI:** 10.3390/life12071061

**Published:** 2022-07-15

**Authors:** Rova Virgana, Nur Atik, Julia Windi Gunadi, Evelyn Jonathan, Dona Erisa Ramadhani, Ray Sebastian Soetadji, Hanna Goenawan, Ronny Lesmana, Arief Kartasasmita

**Affiliations:** 1 Department of Ophthalmology, Faculty of Medicine, Universitas Padjadjaran, Professor Eyckman 38, Bandung 40161, Indonesia; a.kartasasmita@unpad.ac.id; 2 Cicendo National Eye Hospital, Cicendo 4, Bandung 40117, Indonesia; 3 Biology Cell Division, Department of Biomedical Sciences, Faculty of Medicine, Universitas Padjadjaran, Raya Bandung-Sumedang Km 21, Bandung 45363, Indonesia; n.atik@unpad.ac.id; 4 Department of Physiology, Faculty of Medicine, Maranatha Christian University, Surya Sumantri 65, Bandung 40164, Indonesia; julia.windi@maranatha.ac.id; 5 Faculty of Medicine, Maranatha Christian University, Surya Sumantri 65, Bandung 40164, Indonesia; 1910008@maranatha.ac.id (E.J.); 1910140@maranatha.ac.id (D.E.R.); 1810095@maranatha.ac.id (R.S.S.); 6 Physiology Cell Division, Department of Biomedical Sciences, Faculty of Medicine, Universitas Padjadjaran, Raya Bandung-Sumedang Km 21, Bandung 45363, Indonesia; hanna@unpad.ac.id (H.G.); ronny@unpad.ac.id (R.L.); 7 Physiology Molecular Laboratory, Biological Activity Division, Central Laboratory, Universitas Padjadjaran, Raya Bandung-Sumedang Km 21, Bandung 45363, Indonesia

**Keywords:** diabetic retinopathy, MitoTEMPOL, mitochondria-targeted antioxidant, retinal vascularization, autophagy, apoptosis

## Abstract

Diabetic retinopathy leads to retinal malfunction, blindness, and reduced quality of life in adult diabetes patients. The involvement of reactive oxygen species (ROS) regulation stimulated by high blood glucose levels opens the opportunity for ROS modulator agents such as MitoTEMPOL. This study aims to explore the effect of MitoTEMPOL on ROS balance that may be correlated with retinal vascularization pattern, autophagy, and apoptosis in a streptozotocin-induced rat model. Four groups of male Wistar rats (i.e., control, TEMPOL (100 mg/kg body weight [BW]), diabetic (streptozotocin, 50 mg/kg BW single dose), and diabetic + TEMPOL; *n* = 5 for each group) were used in the study. MitoTEMPOL was given for 5 weeks, followed by funduscopy, and gene and protein expression were explored from the rat’s retina. Streptozotocin injection decreased bodyweight and increased food and water intake, as well as fasting blood glucose. The results showed that MitoTEMPOL reduced retinal vascularization pattern and decreased superoxide dismutase gene expression and protein carbonyl, caspase 3, and caspase 9 protein levels. A modulation of autophagy in diabetes that was reversed in the diabetic + TEMPOL group was found. In conclusion, MitoTEMPOL modulation on autophagy and apoptosis contributes to its role as a potent antioxidant to prevent diabetic retinopathy by inhibiting ROS-induced retinal vascularization patterns.

## 1. Introduction

Diabetes mellitus is a major health problem in both developing and developed countries [1]. The estimation of the global prevalence of diabetes mellitus worldwide was 9.3% (463 million) in 2019, which was projected to grow to 10.2% (578 million) in 2030 [2]. In the list of countries with the highest estimated cases of diabetes in 2030, Indonesia was the fourth after India, China, and the USA, with ~8.4 million in 2000 and projected to be 21.3 million in 2030 [3]. As a chronic disease, diabetes mellitus may induce macrovascular and microvascular complications, including diabetic retinopathy [4].

The incidence of diabetic retinopathy was 126.6 million and is estimated to increase to 191.0 million by 2030 [5]. A study conducted in Jogjakarta, Indonesia, found that the prevalence of diabetic retinopathy was 43.1% in adults >30 years of age with type 2 diabetes mellitus, with 4% bilateral blindness due to diabetic retinopathy [6]. As a major microvascular complication of diabetes mellitus, diabetic retinopathy is the leading cause of blindness in adults with diabetes mellitus, with the incidence of its occurrence reaching 90% after 25 years of having diabetes mellitus [7,8]. Diabetic retinopathy is considered a major microvascular complication of diabetes mellitus and a leading cause of blindness.

The gold standard for diabetic retinopathy (DR) treatment is laser photocoagulation therapy, but 50% of patients still undergo retinopathy progression even after receiving the therapy [9]. Recent studies have shown better efficacy and functional improvement of DR when laser photocoagulation is combined with pharmacotherapy [10]. Pharmacologic treatment choices for DR are antivascular endothelial growth factors (VEGF; pegaptanib, bevacizumab, and ranibizumab), corticosteroids, and other agents involved in biochemical pathways (e.g., antioxidants) [9]. Compared to laser photocoagulation therapy, the anti-VEGF has a very short effect and is not qualified to be considered as a gold standard for DR treatment [11]. Consequently, the combination of anti-VEGF and photocoagulation laser has been proven to be more effective for patients with DR [12]. Although this is recommended, the laser could decrease the patient’s color vision, peripheral vision, and night vision, inducing scars that may contribute to chorioretinal atrophy [13]. Therefore, preventing the progression of DR from its earliest stage is very important, and this prevention could be elaborated by giving a potent antioxidant, considering the imbalance of reactive oxygen species (ROS) which act as a trigger of oxidative stress and cell death in diabetic complication [14].

Mitochondria-targeted antioxidants (MTA) are antioxidants that are directly connected with the mitochondria as the target and are specifically accumulated there to protect the targeted tissues [15]. MitoTEMPOL is an MTA that helps the mitochondria to clear superoxide [16]. It has a nitroxide conjugated with TPP moiety that amplifies its potential role against oxidative stress in the setting of many pathological conditions, including diabetic complications in the cardiovascular system [17,18,19]. According to DR pathogenesis, MitoTEMPOL as a mitochondria-targeted antioxidant could serve as an alternative treatment for that disease.

Molecular mechanisms that may elucidate the MitoTEMPOL mechanism against oxidative stress are the changes of superoxide dismutase (SOD) and protein carbonyl that signify the level of oxidative stress, cell death denoted by the modulation of caspase activities, and survival indicated by autophagy modulation. A recent study showed that TEMPOL inhibited carbonyl formation in platelet and plasma proteins [20], reduced podocyte apoptosis in experimental diabetes mellitus [21] and inhibited hepatic apoptosis in acetaminophen induced acute hepatotoxicity [22], and reversed atherosclerosis through autophagy restoration [23]. Nevertheless, studies about the molecular mechanism of MitoTEMPOL in the early stage of DR complication is still limited. Thus, understanding these mechanisms is crucial to finding ways to prevent blindness starting from the earliest stage of the disease.

## 2. Materials and Methods

### 2.1. Housing and Handling of the Animals

Twenty-four male, 6–8 weeks old Wistar rats were obtained from Biofarma, Bandung, Indonesia. After 2 weeks of environmental adaptation, the rats were then divided into four groups (i.e., control, TEMPOL, diabetic, and diabetic + TEMPOL). The TEMPOL group was administered 100 mg MitoTEMPOL/kg body weight (BW), and the specific dose was counted for each rat. The streptozotocin (STZ) group was treated with streptozotocin at a dose of 50 mg/kg BW, while the STZ + TEMPOL was treated with TEMPOL 100 mg/kg BW and streptozotocin at a dose of 50 mg/kg BW, as derived based on the human-to-rat dose conversion. All procedures were performed in studies involving animals based on the use and care of laboratory guidelines. The rats were given a standard chow diet and housed at room temperature with 12 h of light and dark cycles every day. The body weight of experimental animals was also measured before and every week after the STZ injection [24].

### 2.2. MitoTEMPOL and STZ Dose

MitoTEMPOL was purchased from Sigma-Aldrich Co. (Saint Louis, MO, USA), and STZ was purchased from Cayman Chemical Co. (Ann Arbor, MI, USA). In addition, 100 mg/kg of MitoTEMPOL was used for each rat, peroral, five times a week for 5 weeks, while STZ in DMSO and sterile MilliQ was given via peritoneal injection, 50 mg/kg BW single dose. The MitoTEMPOL and STZ doses were based on previous studies [25,26]. After 5 weeks, the animals were sacrificed, the retina was rapidly excised and weighed, and the retina was taken. Two sets of experiments were conducted: one for RNA extraction continued with quantitative polymerase chain reaction (PCR), and the other was for protein extraction continued with Western blot.

### 2.3. Fundus Photography and Average Numbers of Retinal Vessel

The tools used to take fundus photographs of Wistar Rat were iPhone 7 Plus^®^ (Los Altos, CA, USA), Volk Digital Widefield^®^ (Mentor, OH, USA), Xyla Injection (50 mL), Xylazine 2% injection, Ivanes Ketamine HCL IV/IM injection (1000 mg/10 mL), and Spuit needle 26 G. The first step to take a fundus photograph was to prepare the setting in a room with enough light that was evenly spread above the working desk so no scattered or light shadow will be observed. Each rat was given codes following animal sampling and anesthetized through a cocktail intramuscular injection of xylazine/ketamine in the rat’s thigh with 10 mg/(200 g body weight; bw) ketamine in combination with 1 mg/(200 g bw) xylazine. Thereafter, the rats were anesthetized after 5 min.

The next step was to hold the rat to the side so the rat’s eyes are facing up and holding the widefield lens in front of the rat’s eye at about 5 to 10 mm. The phone camera was positioned in front of the lens at about 5 cm, moving it up and down to adjust the best fundus focus (setting of the wide camera, 28 mm f 1.8, 7–12 MP, 1920 × 3412, ISO 50-200, 36 mm, 0 ev, f1.8, and 1/50 s). The fundus picture was captured with a video module for about 5 s (setting of 1080p; 1080 × 1920; 30 fps), captured for two to three shots, without using the flash. Similar steps were performed for the opposite eye, and the photo and video results were checked.

### 2.4. Fasting Blood Glucose

The blood glucose level of the rats was taken from the tail vein before and every week after the injection of STZ until the end of the study. Diabetic rats were characterized by fasting blood glucose level ≥200 mg/dL [27].

### 2.5. Real-Time PCR Studies

According to the manufacturer’s instructions, total RNA extraction of the retina was done with a TRIsure (Bioline, London, UK). The purity and concentration of the extracted RNA were measured by a Multimode Microplate Reader on 268/280 nm absorbance spectrophotometry (M200 Pro, Tecan, Morgan Hill, CA, USA). A quantitative PCR was performed using My Taq™ One-Step RT-PCR Kit (Bioline, London, UK) following the manufacturer’s instructions. GAPDH levels were taken for normalization, and fold change was calculated using 2^−^^ddCt^. The order of primers that were applied for this experiment is shown in Table 1.

### 2.6. Western Blot Analysis

Retinal tissues were dissected from the rats, weighed, and lysed in cold RIPA lysis buffers with protease and phosphatase inhibitors. The samples were centrifuged and combined with sample buffers (containing beta-mercaptoethanol) followed by heat denaturation processes (95 °C) for 5 min and directly placed on ice for snap freezing. Samples in equal amounts (10 μg) were electrophoresed in an SDS-PAGE gel for 20 min at 80 V and continued for 60 min at 120 V. In addition, the gel was blotted to a nitrocellulose membrane for 60 min at 200 mA. The membranes were then blocked for 24 h using a 0.75% blocking skim milk in a Tris-buffered with 0.1% Tween 20. In addition, membranes were incubated using a goat polyclonal caspase3 (#AF-605-NA; R & D Systems, Minneapolis, MN, USA) and goat polyclonal caspase9 (#AF8301; R & D Systems) mouse monoclonal Beta Actin (MA5-15739; R & D Systems,). The primary antibody dilution ratio is 1:1000. The secondary antibody was purchased from LICOR Infrared System, and the dilution for the secondary antibody is 1:15,000 (LiCOR Odyssey Clx Chemiluminescent and Secondary Antibody-infrared System, Lincoln, NE, USA). The band intensities were determined using the LiCOR Quantification Software. Each blot was stripped by the stripping buffer from Thermo Scientific (Waltham, MA, USA) following the recommended procedures and re-probed by an internal control primary antibody, beta-actin, as an internal control to monitor the integrity of the protein.

For the protein carbonyl assay experiment, the protocol followed the manufacturer’s recommendation (#ab170820; Abcam, Waltham, MA, USA). The protein carbonyl expression was detected by a chemiluminescence reagent (GE Healthcare, Chicago, IL, USA), (LI-COR C-DiGit Chemiluminescence Western Blot Scanner, Lincoln, NE, USA).

### 2.7. Statistical Analysis

Data obtained in this study were presented as mean ± SEM. The results were analyzed using a one-way analysis of variance test/Kruskal–Wallis, followed by LSD/Mann–Whitney for post hoc test.

Statistical analysis was performed using SPSS software 20.0 (Armonk, NY, USA). The level of significance test was fixed at *p* < 0.05.

## 3. Results

### 3.1. Effect of STZ Injection and MitoTEMPOL on Body Weight, Fasting Blood Glucose Level, Food Intake, and Water Intake

**Figure 1:**
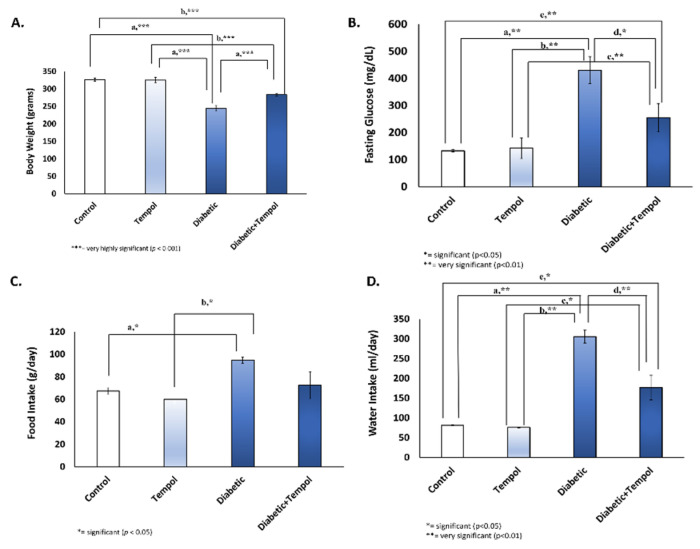
Effect of STZ injection and MitoTEMPOL on body weight, fasting blood glucose level, food, and water intake. (**A**) STZ injection decreased body weight in the diabetic compared with the control (a, *p* = 0.000), TEMPOL (a, *p* = 0.000), and diabetic + TEMPOL (a, *p* = 0.000) groups and in the diabetic + TEMPOL group compared with the control (b, *p* = 0.000) and TEMPOL (b, *p* = 0.000) groups. (**B**) STZ injection also increased fasting blood glucose level in the diabetic compared with the control (a, *p* = 0.008), TEMPOL (b, *p* = 0.008), and diabetic + TEMPOL (d, *p* = 0.028) groups and in the diabetic + TEMPOL group compared with the control (c, *p* = 0.008) and TEMPOL (e, *p* = 0.008) groups. (**C**) An increase in food intake was found in the diabetic group compared with the the control (a, *p* = 0.038) and diabetic group compared with TEMPOL (b, *p* = 0.017). (**D**) We found an increase in water intake in the diabetic compared with the control (a, *p* = 0.001), TEMPOL (b, *p* = 0.001), and diabetic + TEMPOL (d, *p* = 0.006) groups and in the diabetic + TEMPOL group compared with the control (e, *p* = 0.018) and TEMPOL (c, *p* = 0.015) groups.

Single dose STZ injection decreased body weight in the diabetic group compared with the control (a, *p* = 0.000), TEMPOL (a, *p* = 0.000), and diabetic + TEMPOL (a, *p* = 0.000) groups and in the diabetic + TEMPOL group compared with the control (b, *p* = 0.000) and TEMPOL (b, *p* = 0.000) groups (Figure 1A). STZ injection also increased fasting blood glucose level in the diabetic group compared with the control (a, *p* = 0.008), TEMPOL (b, *p* = 0.008), and diabetic + TEMPOL (d, *p* = 0.028) groups and in the diabetic + TEMPOL group compared with the control (c, *p* = 0.008) and TEMPOL (e, *p* = 0.008) groups (Figure 1B). This study also found an increase in food intake (Figure 1C) in diabetic compared with the control (a, *p* = 0.038) and TEMPOL (b, *p* = 0.017);and water intake (Figure 1D) in the diabetic compared with the control (a, *p* = 0.001), TEMPOL (b, *p* = 0.001), and diabetic + TEMPOL (d, *p* = 0.006) groups and in the diabetic + TEMPOL group compared with the control (e, *p* = 0.018) and TEMPOL (c, *p* = 0.015) groups.

### 3.2. Effect of STZ Injection and MitoTEMPOL on Funduscopy and Vessel Diameter of the Retina

**Figure 2:**
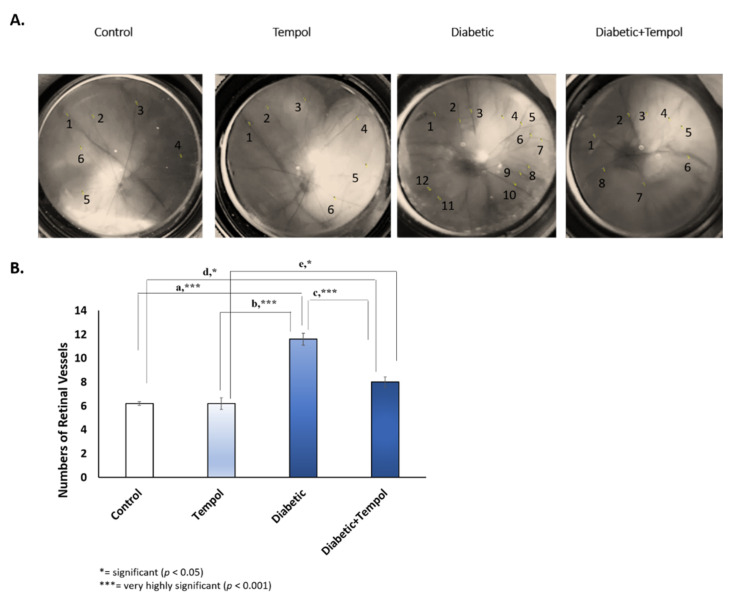
Effect of STZ injection and MitoTEMPOL on funduscopy of the retina and numbers of retinal vessels. (**A**) Representative fundus photography for each group was used in this study. Retinal vascularization pattern increased in rats retinal after the injection of single dose STZ. (**B**) Average numbers of retinal vessel showed increased retinal vascularization pattern in the diabetic group compared with the control (a, *p* = 0.000), TEMPOL (b, *p* = 0.000), and diabetic + TEMPOL (c, *p* = 0.000) groups and in the diabetic + TEMPOL group compared with the control (d, *p* = 0.024) and TEMPOL (e, *p* = 0.024) groups.

The vascularization pattern in the retinal vessel was found in the diabetic group and compared with the control (a, *p* = 0.000), TEMPOL (b, *p* = 0.000), and diabetic + TEMPOL (c, *p* = 0.000) groups and in the diabetic + TEMPOL group compared with the control d, (*p* = 0.024) and TEMPOL (e, *p* = 0.024) groups. These changes are shown in Figure 2.

### 3.3. Effect of STZ Injection and MitoTEMPOL on SOD and Autophagy Gene Expression

**Figure 3:**
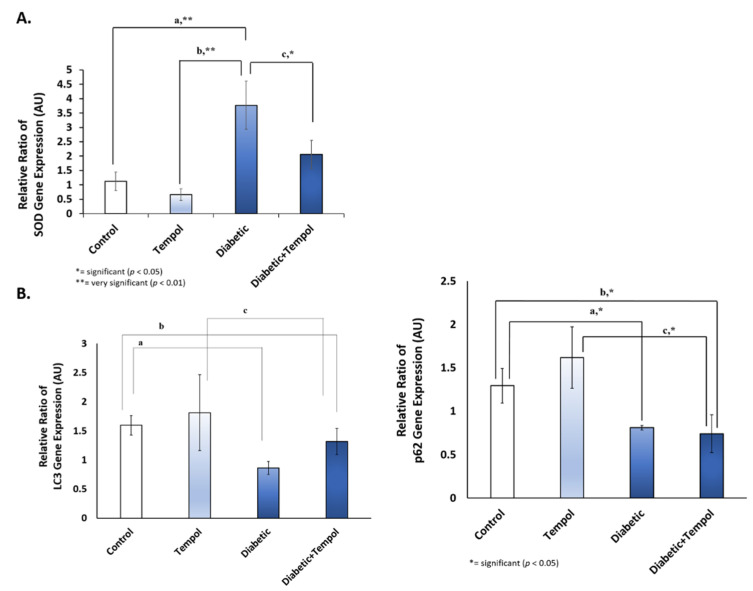
Effect of STZ injection and MitoTEMPOL on SOD and autophagy gene expression. (**A**) Relative ratio of SOD gene expression increased in the diabetic group compared with the control (a, *p* = 0.002), TEMPOL (b, *p* = 0.001), and diabetic + TEMPOL (c, *p* = 0.034) groups. (**B**) The relative ratio of LC3 gene expression was lower in the diabetic group compared with the other groups, while p62 gene expression decreased in the diabetic group compared with the control group (a, *p* = 0.023) and the diabetic + TEMPOL group compared with the control (b, *p* = 0.016) and TEMPOL (c, *p* = 0.044) groups.

STZ injection and MitoTEMPOL modulated SOD and autophagy gene expression (Figure 3). SOD gene expression was found to be increased in the diabetic group compared with the control (*p* = 0.002), TEMPOL (*p* = 0.001), and diabetic + TEMPOL (*p* = 0.034) groups. As for autophagy gene expression, the relative ratio of LC3 gene expression was lower in the diabetic group compared with the other groups, while p62 gene expression decreased in the diabetic group compared with the control (*p* = 0.023), diabetic + TEMPOL compared with the control (*p* = 0.016) and TEMPOL (*p* = 0.044) groups.

### 3.4. Effect of STZ Injection and MitoTEMPOL on Carbonyl and Caspase Protein Levels

STZ injection and MitoTEMPOL modulated carbonyl and caspase protein levels. The high carbonyl protein level in the diabetic group was higher compared with the TEMPOL and diabetic + TEMPOL groups. STZ injection also increased cleaved caspase 3 protein levels in the diabetic group compared with the control (*p* = 0.024), TEMPOL (*p* = 0.025), and diabetic + TEMPOL (*p* = 0.016) groups and increased cleaved caspase 9 in the diabetic group compared with the control, TEMPOL, and diabetic + TEMPOL (*p* = 0.001) groups.

## 4. Discussion

Under pathological conditions of diabetes mellitus, an imbalance may exist between the production and destruction of ROS due to increased ROS production, decreased clearance, or both [32]. This ROS imbalance could induce damage to macromolecules—proteins, lipids, and DNA [33]—and trigger oxidative stress and cell death as a diabetic complication, including in the retina [14]. In DR, ROS increase is caused by the inactivation of complex III and activation of matrix metalloproteinase, which leads to damage to retinal mitochondria and finally induces mitochondrial dysfunction [7,34,35]. The prevention of mitochondrial damage could be facilitated by a potent antioxidant that targeted the mitochondria known as MTA [36]. In this study, MitoTEMPOL was used as an MTA because its potent antioxidant properties have been proven in previous studies [18,37,38].

MTA plays a critical role in decreasing the production of ROS during mitochondria malfunction by specifically entering the mitochondria [15]. Physiologically, the mitochondria produce ROSs and ATPs, as well as scavenging free radicals that lead to mitochondrial redox homeostasis (MRH). However, the MRH will be negatively affected due to ROS increase and ATP decrease and scavenging if the mitochondria are malfunctioning [39]. This situation results in the development of related diseases. The ROS surge causes the cell to have an insufficient amount of antioxidants to perform the scavenging [15]. Due to these complications, several studies focus on solving the correct amount of exogen antioxidants for the targeted mitochondria and the redox homeostasis to reverse the mitochondrial malfunction [40]. A previous study showed that the ROS increase occurred 15 days after STZ injection, inducing repair of damaged mitochondria at 2 months. However, this mechanism only lasted until 6 months, when mitochondrial numbers started to decrease: a sign that MRH was disturbed [28].

STZ injection caused diabetes mellitus, which may be due to the destruction of β-cells of the islets of Langerhans of the pancreas. STZ-induced diabetes is characterized by severe loss in body weight caused by the degradation of structural proteins, which are responsible for the changes in body weight [41]. This model provides a relevant example of endogenous chronic oxidative stress due to the resulting hyperglycemia [42]. Single-dose STZ injection in the rat (50 mg/kg BW) induces the necrosis of β cell pancreas in a rapid and irreversibly manner [43], and this causes elevated fasting blood glucose (hyperglycemia) and symptoms of diabetes mellitus (polyuria, polydipsia, polyphagia, fatigue, gastric dysfunction, and unexplained weight loss) [44,45]. In diabetes mellitus, blood glucose cannot enter cells, and if the energy supply in cells is insufficient, the brain’s hunger center is stimulated, resulting in a considerable increase in the number of eating times and food intake, leading to an increase in blood glucose level [46]. The inability of glucose to enter energy metabolism, as well as structural protein loss, may explain the weight loss in diabetics [47]. In this study, the rats induced by STZ showed a decrease in body weight (Figure 1A), an increase in fasting blood glucose (Figure 1B), and an increase in food, water intake, and increased urination proven by the wet bedding (Supplementary Figure S1), but the administration of MitoTEMPOL could reverse them (Figure 1).

DR progression depends on the severity of induced hyperglycemia and diabetes duration and causing a decrease in tissue oxygenation, resulting in ischemia. It is usually asymptomatic at the beginning, slowly progressing in time, but an increase in intraretinal hemorrhage size would be found in preproliferative DR [48]. One of the main causes of new vessel creation in retinal proliferative disease is an ischemia-induced release of cytokines (e.g., VEGF into the vitreous cavity) [49]. Neovascularization involved networks of retinal vessels on the retina’s surface that extends to the vitreous cavity, and this clinical condition would normally spot on a slit lamp examination but may be a missed diagnosis in the early stage [50]. As the main characterization of proliferative DR, neovascularization increases hemorrhage, glaucoma, and retinal detachment, finally resulting in loss of vision [51]. In this study, an increase in retinal vascularization pattern after a single dose of STZ injection was found (Figure 2), as shown by the funduscopy photograph (Figure 2A) and the average number of the retinal vessel (Figure 2B). Moreover, MitoTEMPOL decreased the retinal vascularization pattern caused by STZ injection (Figure 2).

The antioxidant properties of MitoTEMPOL may also modulate SOD gene expression and the carbonylation of protein. SOD is an antioxidant to protect against oxidative stress, which is important for the body. In addition, it acts as an anti-inflammation agent to confront the stress and prevent further damage to the cell [52]. The SOD’s function is to avert excess ROS production [53]. An increase in SOD gene expression in the diabetic group was found compared with the other groups (Figure 3A), suggesting an increase in antioxidants as an attempt to protect the retinal cells from oxidative stress caused by hyperglycemia. This result is consistent with a study that concluded that the growth of SOD in the diabetic group may be related to a higher glucose intercellular concentration that activates the hexosamine metabolism and aldose reductase paths, resulting from ROS increase [54]. MitoTEMPOL may also modulate antioxidant levels as it efficiently inhibited mitochondrial superoxide generation in high-glucose-stimulated cardiomyocytes, and its administration may have reduced the antioxidant levels and have therapeutic benefit in diabetic cardiac complications [18]. Failure in insulin formation due to the STZ injection will degrade the GLUT1 expression on the retina’s endothelial cell, hexosamine metabolism, and aldose reductase [25,55]. A recent study about reciprocal effects of oxidative stress on heme oxygenase expression activity demonstrates that chronic oxidative stress seen in SOD has reciprocal effects on heme oxygenase (HO) expression and activity [56].

**Figure 4:**
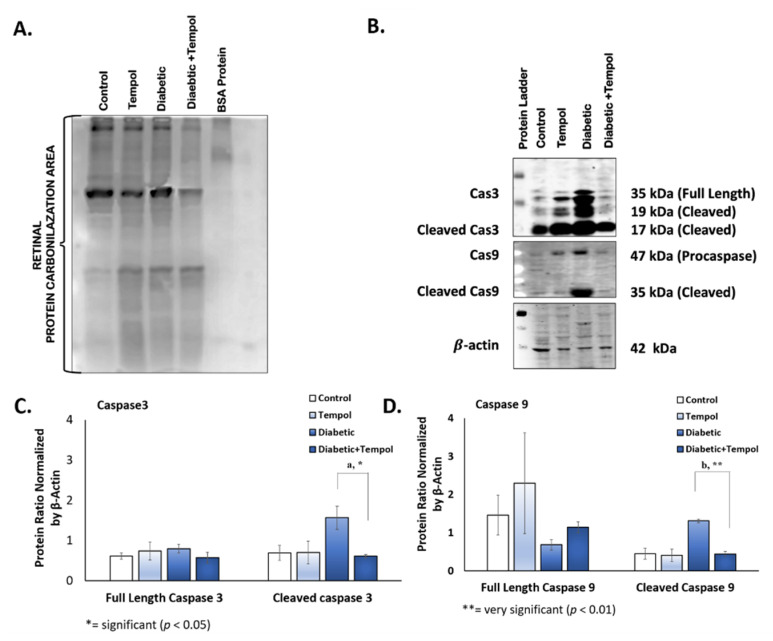
Effect of STZ injection and MitoTEMPOL on carbonyl and caspase protein levels. (**A**) Protein carbonylation was increased in the diabetic group compared with the other groups. (**B**) Representative protein immunoblot of caspase 3, cleaved caspase 3, caspase 9, and cleaved caspase 9 protein. (**C**) Cleaved caspase 3 protein level was significantly reduced in the diabetic + TEMPOL group compared to the diabetic group only. (**D**) Cleaved caspase 9 protein level was significantly reduced in the diabetic + TEMPOL group compared to the diabetic group only.

Carbonylation protein is the process of producing adduction lipid–protein that is common in various tissues due to acute or chronic oxidative stress, which has a cause-and-effect relationship with metabolic dysfunction [57]. Previous studies have shown an increase in protein carbonylation in red blood cells and eyes of proliferative DR patients [58,59]. In the recent study about superoxide dismutase mimetic tempol and its treatment in rat skeletal muscle capillaris, it was found that tempol lowered protein carbonylation [60]. Consistent with those studies, the results of the current study indicate that protein carbonylation levels in the diabetic group are higher compared with the other groups, and MitoTEMPOL decreased this protein carbonylation (Figure 4).

Autophagy is a survival mechanism for the preservation of organismal homeostasis [61]. P62 is an autophagy receptor that depends on the interaction with ATG8, which is critical for selective autophagy. ATG8 belongs to the LC3 protein family that is considered to have a similar function [62,63]. In DR, abnormal autophagy plays an important role in its molecular mechanism, and excessive oxidative stress may induce pericyte cell death, which is important in the structure and function of retinal capillary [64]. The sophisticated connection between autophagy and apoptosis is also responsible for determining the severity of cellular apoptosis and DR progression [65,66]. Oxidative stress and endoplasmic reticulum stress in diabetes mellitus cause mitochondrial damage that eventually stimulates Fas and TNFα as dearth receptors to set off apoptosis [64]. Rodent models of DR showed that retinal capillary cell apoptosis precedes the appearance of degenerative capillaries and pericyte ghosts [67,68]. In this study, the P62 and LC3 were analyzed to have declined in the diabetic group, denoting an alteration in autophagy gene expression that may indicate excessive autophagy, while an increase in the LC3 gene expression in the diabetic + TEMPOL group may be correlated to the role of MitoTEMPOL in balancing ROS production that maintains the homeostatic process. The modulation of autophagy gene expression indicated that autophagy activity may be stimulated by MitoTEMPOL treatment, but further study needs to be conducted to confirm this hypothesis. An increase in caspase 3 and caspase 9 protein levels in the diabetic group compared with the diabetic + TEMPOL group was also found, indicating the role of MitoTEMPOL in reducing apoptosis in the retinal cell after STZ injection.

## 5. Conclusions

The pathogenesis of DR involves oxidative and endoplasmic reticulum stress resulting in mitochondrial damage. At its earliest stage, MitoTEMPOL, as a potent antioxidant, may have a beneficial role in inhibiting retinal vascularization patterns as a result of mitochondrial damage in the retina. Taken together, STZ induces hyperglycemic-stimulated ROS levels in the retina, which is responsible for retinal neovascularization in the early stage of DR. ROS modulation by MitoTEMPOL could potentially reserve the retinal function by activating LC3 and antioxidant enzyme (SOD) gene expression and inhibiting protein carbonylation and apoptosis. Thus, the utilization of MitoTEMPOL may be beneficial for future clinical application in the early onset of diabetes mellitus complications in the retina.

## Figures and Tables

**Table 1:** Primers Used for Real-time PCR.

Gene Symbol	Primer Sequence (5′ to 3′) Upper Strand: Sense Lower Strand: Antisense	Product Size (bp)	References
SOD2	AATGTTGTGTCGGGCGGCGT	173 bp	[28]
	AGGTCGCGTGGTGCTTGCTG		
LC3	CATGCCGTCCGAGAAGACCT	70 bp	[29]
	GATGAGCCGGACATCTTCCACT		
p62	CGGAAGTCAGCAAACC	149 bp	[30]
	ATGCGTCCAGTCGTCA		
GAPDH	AGGTCGGTGTGAACGGATTTG	123 bp	[31]
	TGTAGACCATGTAGTTGAGGTCA		

## Data Availability

Not applicable.

## Supplementary Materials

The following supporting information can be downloaded at: https://www.mdpi.com/article/10.3390/life12071061/s1, Figure S1: Bedding Appearance from Every Group.
