# Supplementary figures and images for: MitoTEMPOL Inhibits ROS-Induced Retinal Vascularization Pattern by Modulating Autophagy and Apoptosis in Rat-Injected Streptozotocin Model
Source: Life (Basel). 2022 Jul 15;12(7):1061. doi: 10.3390/life12071061 (PMC9320075; doi:10.3390/life12071061)

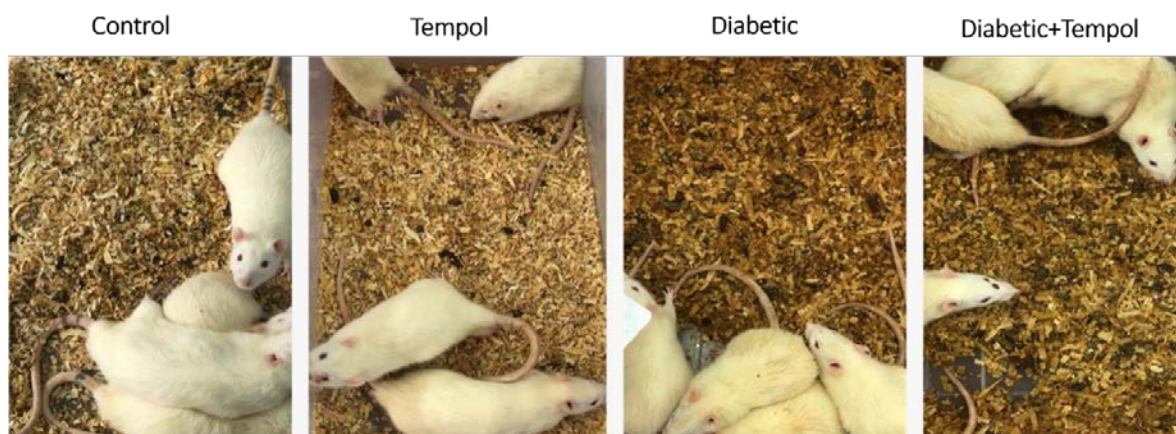

**Supplementary Figure S1.** Bedding Appearance from Every Group.

Supplement: Supplementary file 1 [file life-12-01061-s001.zip › life-1780139-supplementary.pdf]
